# Molecularly Targeted Fluorescent Sensors for Visualizing and Tracking Cellular Senescence

**DOI:** 10.3390/bios13090838

**Published:** 2023-08-23

**Authors:** Zhirong He, Kun Xu, Yongming Li, Han Gao, Tingting Miao, Rui Zhao, Yanyan Huang

**Affiliations:** 1 College of Chemistry & Materials Engineering, Wenzhou University, Wenzhou 325035, China; 21451230015@stu.wzu.edu.cn; 2 Beijing National Laboratory for Molecular Sciences, CAS Key Laboratory of Analytical Chemistry for Living Biosystems, CAS Research/Education Center for Excellence in Molecular Sciences, Institute of Chemistry, Chinese Academy of Sciences, Beijing 100190, China; xukun@iccas.ac.cn (K.X.); liyongming@iccas.ac.cn (Y.L.); gaohan20@iccas.ac.cn (H.G.); zhaorui@iccas.ac.cn (R.Z.); 3 School of Chemistry, University of Chinese Academy of Sciences, Beijing 100049, China

**Keywords:** cellular senescence, fluorescent sensors, molecular recognition, targeted imaging

## Abstract

Specific identification and monitoring of senescent cells are essential for the in-depth understanding and regulation of senescence-related life processes and diseases. Fluorescent sensors providing real-time and in situ information with spatiotemporal resolution are unparalleled tools and have contributed greatly to this field. This review focuses on the recent progress in fluorescent sensors for molecularly targeted imaging and real-time tracking of cellular senescence. The molecular design, sensing mechanisms, and biological activities of the sensors are discussed. The sensors are categorized by the types of markers and targeting ligands. Accordingly, their molecular recognition and fluorescent performance towards senescence biomarkers are summarized. Finally, the perspective and challenges in this field are discussed, which are expected to assist future design of next-generation sensors for monitoring cellular senescence.

## 1. Introduction

Cellular senescence, an irreversible state of cell cycle arrest, was firstly described in the 1960s by Hayflick and colleagues [1,2]. In addition to replicative senescence in which the shortening of telomeres is involved, senescence has been revealed to be related to various cellular stresses, such as DNA damage and reactive oxygen species (ROS), and thus is closely associated with a broad range of biological processes and diseases [3,4,5]. With increasing knowledge on the positive and negative aspects of cellular senescence, senescent cells have been recognized as attractive targets for antiaging approaches. Nevertheless, due to the involvement in tumorigenesis and tumor progression, senescent cells are highlighted as targets for the treatment of cancer [6]. To these ends, selective and sensitive detection and tracking of senescent cells has become crucial, where targeted probes are highly demanded.

Considerable research efforts have been devoted to the identification of senescent cells. It has been demonstrated that all senescent cells develop a complex senescent-associated secretory phenotype, which is variable, plastic, and dependent on cell types and senescence inducer [7]. The senescence phenotype is characterized by cell growth arrest, usually with a DNA content typically in the G1 phase [3]. Under stimuli, senescent cells also develop resistance to cell-death signals and altered gene expression. As a result of these signaling pathways, senescent cells exhibit morphologic abnormalities, including cell enlargement, vacuolization, and flattening [8]. Meanwhile, accumulation of lysosomes and mitochondria, and nuclear changes have also been identified during cellular senescence. Aiming at distinguishing senescent cells at the molecular level, markers representing the senescent state have received extensive research interests. The senescence-associated β-galactosidase (SA-β-Gal) is the first marker used for the specific detection of senescent cells. Afterwards, more enzymes and proteins emerged as molecular signatures for the recognition of senescent cells, which also lays the foundation for the design of molecular targeting probes.

Fluorescence imaging is powerful for tracing biomolecules in living systems, providing real-time and in situ information with high spatial and temporal resolution [9,10,11,12]. To achieve this, fluorescent chemo- and bio-sensors, specifically recognizing and interacting with a target of interest to induce fluorescence signals, are indispensable tools [13,14,15]. In the past decade, motivated by the essential roles of senescence in a diverse range of physiological and disease processes, a growing body of fluorescence sensors were constructed for imaging senescence in cells and in vivo. The design principle usually includes the conjugation of affinity ligands such as carbohydrates, peptides, and antibodies, to emissive dyes or nanoparticles. Facilitated by high-affinity and selective recognition, fluorescent signals can be generated to specifically indicate the dynamic changes of senescence markers. These studies provide valuable information for in-depth understanding of the molecular mechanisms of cellular senescence, but also contribute to the development of theranostic strategies for senescence-associated diseases.

In view of the rapid development of fluorescence-based approaches for cellular senescence analysis, we herein summarize recent advances in molecularly targeted fluorescence sensors and their applications in tracking senescence in biological systems. Since there have been several excellent reviews on the detection of cellular senescence [16,17,18], this review focuses on the molecular design and sensing strategies toward specific senescence-related biomarkers. Accordingly, the content is arranged into four subsections based on the types of markers and mechanisms of targeting. The first part is devoted to the introduction of fluorescent probes that target enzymes such as SA-β-Gal due to their well-characterized functions and the large body of emerging research works. For the recognition of non-enzyme molecular targets, antibodies have been widely employed for the development of highly selective sensors for senescence visualization. Meanwhile, peptides with small molecular size, deep penetrability, and flexibility are ideal targeting elements and have been utilized to guide senescence imaging in living biosystems. Thus, two subsections are aimed to summarize existing advances in imaging cellular senescence by using antibodies and peptides as targeting units, respectively. Another attractive progress is the dual-targeting fluorescent sensors which employ two senescence biomarkers to achieve high selectivity and accuracy for detection. We also summarize the research works in this aspect in one subsection. In each subsection, the design principles of the targeted sensors are introduced. The cooperative actions between affinity ligands and emissive moieties are discussed. The sensing performance and bioactivity of the sensors are summarized. Finally, the perspectives and challenges in this field are discussed, which are expected to assist future design of next-generation sensors for monitoring cellular senescence. The four classes of molecularly targeted senescence sensors are catalogized in Table 1 (Please see page 17). In this table, the senescence biomarkers, recognition elements, fluorescent reporters, as well as their sensing performance and applications are summarized, which aims to provide a direct view of recent progress in this field.

## 2. Targeting Enzymes for Senescence Sensing

Senescent cells are metabolically active and sensitive to metabolic states [7]. As one of the major players in cellular metabolism, enzymes undergo significant changes in response to the initiation and progress of senescence, and thus emerge as ideal targets [19]. Among these, SA-β-Gal, which is overexpressed in senescent cells, is the first and most widely used marker for the detection of cellular senescence [20,21]. In recent years, studies from molecular biology have identified more enzymes including hydrolases and oxidases that abnormally accumulate in senescent cells. Based on the highly specific interaction between enzyme and substrate, fluorescent sensors can be rationally designed by incorporating substrate structure to fluorophores. Upon binding and reacting with target enzymes, the fluorescent signals are activated, enabling selective and sensitive indication of the senescence process in living systems.

### 2.1. Sensors Targeting SA-β-Gal

Unlike non-senescent cells whose lysosomal pH is highly acidic (pH 4.5–5.0), the environmental pH in senescent lysosomes increases to pH 6.0, which is not optimal for hydrolytic enzymes. Nevertheless, the remarkably high expression and accumulation of SA-β-Gal in lysosomes enables the efficient enzymatic reaction and thus its detection at a sub-optimal pH (such as pH 6.0) [22,23]. Based on this, synthetic fluorescent probes tethered with β-galactosides as targeting substrates were constructed for sensing catalytic activity of SA-β-Gal [24,25,26,27,28,29]. It was found that both acetylated and non-acetylated galactoses were used as the substrates of SA-β-Gal for the targeted recognition and reaction (Table 1). In one research work that compared the imaging performance of the acetylated and non-acetylated formats of the sensor, it was found that acetylation improved the cell permeability and enabled long-term retention of the sensor inside the cells [30]. Overall, the two types of the probes worked well for the visualization of cellular senescence and can effectively track SA-β-Gal activity in vivo.

**Figure 1:**
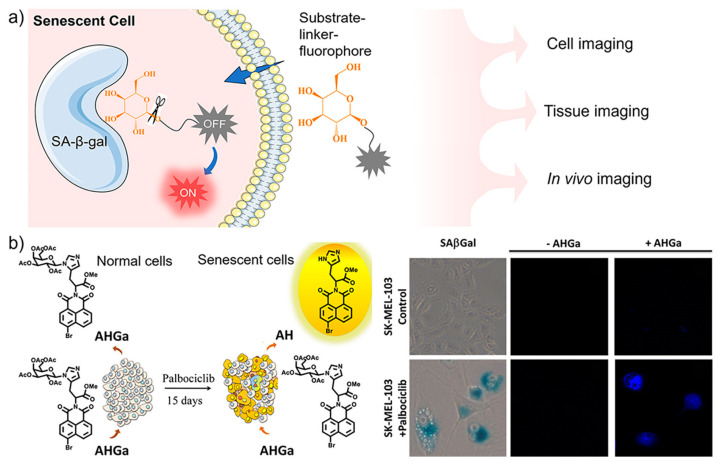
(**a**) Schematic illustration of sensing mechanism of fluorescent sensors with off–on response to SA-β-Gal, whose β-glycosidic bonds are specifically hydrolyzed to release the fluorescence and light up senescent cells; (**b**) An off–on two-photon probe (**AHGa**) was designed for imaging palbociclib-induced senescent cells. Reprinted with permission from [38]. Copyright 2016 American Chemical Society.

Fluorescent off–on probes that are non-emissive in native status and emit strong fluorescence upon target activation provide high signal-to-background signal and thus high sensitivity and resolution for bioimaging [31,32]. Such activatable fluorescence is easy to achieve by enzyme-targeted probes, where the enzymatic reaction can be used as the natural trigger to release fluorescence (Figure 1a). Most of currently available SA-β-Gal-targeting probes act with the switch-on mode to specifically light up senescent cells [33,34,35,36,37]. As a pioneer work, Serrano, Martínez-Máñez, Sancenón and co-workers designed and synthesized an off–on two-photon probe (**AHGa**, Φ_AHGa_ = 0.002) for tracking cellular senescence in vivo [38]. As shown in Figure 1b, the molecular design includes a naphthalimide fluorophore core, an L-histidine methyl ester connector, and an acetylated galactose as a recognition unit. The targeting unit galactose is bonded to an aromatic nitrogen atom of L-histidine via an N-glycosidic bond which can be efficiently hydrolyzed by β-Gal to generate highly emissive **AH** (Φ_AH_ = 0.458, 286-fold enhancement). Targeted imaging of senescent cells with **AHGa** was verified in vitro with human melanoma SK-MEL-103 cells. Palbociclib-induced senescent SK-MEL-103 cells were clearly imaged with remarkable fluorescence enhancement (ca. 10-fold) after treatment with **AHGa**, as compared with control cells under the same treatment. The ability of in vivo tracking senescence was further demonstrated in a tumor-bearing mouse model after the treatment of senescence-inducing chemotherapy.

Compared with conventional one-photon fluorescent probes, two-photon imaging offers advantages of deep tissue penetration, minimal photo-damage, and low background interference [39]. Guided by the above work, a new two-photon naphthalimide-styrene fluorophore was reported by the same group [40]. By targeting lysosomal β-Gal activity, the probe can sensitively detect cellular senescence in various cell lines. The successful fluorescence imaging of senescence sites in cancer and renal fibrosis mouse models indicates the potential of the probe for senescence analysis in aged or damaged tissues.

**Figure 2:**
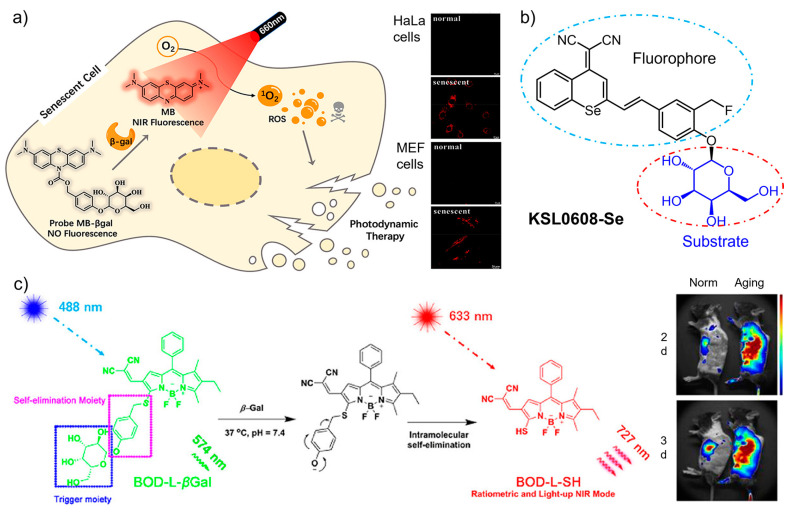
(**a**) A dual-functional β-Gal-activatable probe (**MB-βgal**) was engineered for imaging and photodynamic eliminating senescent cells in situ. Reprinted with permission from [44]. Copyright 2022 American Chemical Society; (**b**) A smart sensor (**KSL0608-Se**) integrating β-Gal targeting and covalent anchoring with photodynamic therapy was designed for the precise tracking and ablation of senescent cells. (**c**) The sensing mechanism of the near-infrared probe (**BOD-L-βGal**) and its incorporation into polymer nanoparticles to imaging senescent cells in vivo. Reprinted with permission from [49]. Copyright 2020 American Chemical Society.

Fluorescent probes operated in the near infrared (NIR) region are highly desirable for in vivo bioimaging owing to the deep penetrability and biocompatibility of the long-wavelength light [41,42]. By introducing fluorophores with NIR absorption and emission to the substrate units, SA-β-Gal-targeting probes have been developed for monitoring cellular senescence [43,44,45,46]. Recently, a dual-functional β-Gal-activatable probe (**MB-βgal**) was engineered for imaging and eliminating senescent cells [44]. As shown in Figure 2a, the modification of β-D-galactopyranoside with methylene blue (MB) provides the probe with high fluorescence quantum yield in the NIR region and ROS-generating activity in the therapeutic window (650–800 nm). To silence the fluorescence in non-targeting status, the 10-N position of the MB skeleton is caged with a carbamate group which is further linked with β-D-galactopyranoside. With this design, the cleavage by β-Gal releases free MB to emit NIR signal for senescence imaging in situ. Meanwhile, the production of singlet oxygen under irradiation selectively eliminates senescent cells. Zhang and co-workers created a new class of dual-state luminophores for the functionalization of β-galactoside. The designed sensors displayed tunable spectra, a large Stokes shift (>170 nm), and NIR emission, which enabled highly selective sensing of SA-β-Gal in senescent cells and liver metastasis with high contrast [47]. More recently, a dual-modal fluorescent/photoacoustic imaging sensor was developed [48], in which β-D-galactose was designed as the specific substrate for SA-β-Gal, and biotin as the tumor targeting element. It is interesting to find that the incorporation of biotin not only accelerated the reaction kinetics, but also helped the cellular uptake of the sensor into tumor cells. The dual-modal imaging provided high sensitivity for real-time monitoring cellular senescence in vivo.

More recently, a smart sensor (**KSL0608-Se**) integrating β-Gal targeting and covalent anchoring with photodynamic therapy was developed for precise tracking and ablation of senescent cells [50]. As shown in Figure 2b, as the substrate of SA-β-Gal, a β-D-galactosyl group was modified with a selenium (Se)-containing fluorescence tag. The attachment of a fluoromethyl moiety to the framework provides the sensor with self-immobilizing ability. In the presence of SA-β-Gal, **KSL0608-Se** is selectively activated to form a bioorthogonal receptor which further reacts with surrounding proteins to form NIR-emitting products. With the off–on NIR signal, the sensor was found to anchor senescent cells with single-cell resolution. Compared with the analogue sensor, the replacement of the O atom with Se not only shifts the emission toward the long-wavelength region (721 nm), but also causes singlet oxygen generation upon photo irradiation. Benefitting from the specific recognition with SA-β-Gal and photocontrollability, highly selective imaging and eliminating senescent cells were successfully achieved by **KSL0608-Se** in aged mice.

The combination of small-molecular probes with nanoparticles usually creates favorable properties to facilitate biological applications [51,52]. Gu, Hu, and co-workers incorporated a small molecule-based NIR probe (**BOD-L-βGal**) into polymer nanoparticles (poly(lactic-co-glycolic) acid) to enhance the uptake of the probe by vascular cells and reduce the clearance in the body (Figure 2c) [49]. By this design, the **BOD-L-βGal** containing a β-galactose residue and a boron dipyrromethene fluorophore was efficiently delivered to the target site in mice to realize in vivo ratiometric imaging of senescent cells and vasculature in atherosclerosis. As a class of inorganic nanomaterial, mesoporous silica nanoparticles show distinct advantages as carriers of dyes and drugs due to their high porosity, functionality, and biocompatibility [53,54,55,56]. By loading Nile blue (NB), an organic dye approved by the Food and Drug Administration, into mesoporous silica nanoparticles, Martínez-máñez and Sancenón fabricated a nanocomposite for detecting cellular senescence in vivo [57]. The capping hexagalacto-saccharide on the surface of silica nanoparticles was designed as the trigger with response to SA-β-Gal. Upon hydrolysis, the encapsulated NB was controllably released to produce a marked NIR emission. The off–on signal enabled selective tracing of senescent cells in mouse models.

### 2.2. Sensors Targeting Other Hydrolases and Oxidases

As research works on cellular senescence accumulated, more markers have been discovered whose expression levels are closely related with the progress of senescence. Among these molecular markers, many are lysosomal hydrolases such as α-L-fucosidase (α-fuc), sialidase, and exoglycosidases, due to the abnormal increase of lysosomes in senescent cells [58,59,60,61]. In addition to the lysosome, the mitochondrion is another organelle sensitively responding to the occurrence of senescence [62,63]. As a result, several enzymes in mitochondria were also identified as markers for cellular senescence. Fluorescent sensors designed and synthesized by using these enzymes as the targets have shown specific responses for imaging-guided investigation of senescent cells.

**Figure 3:**
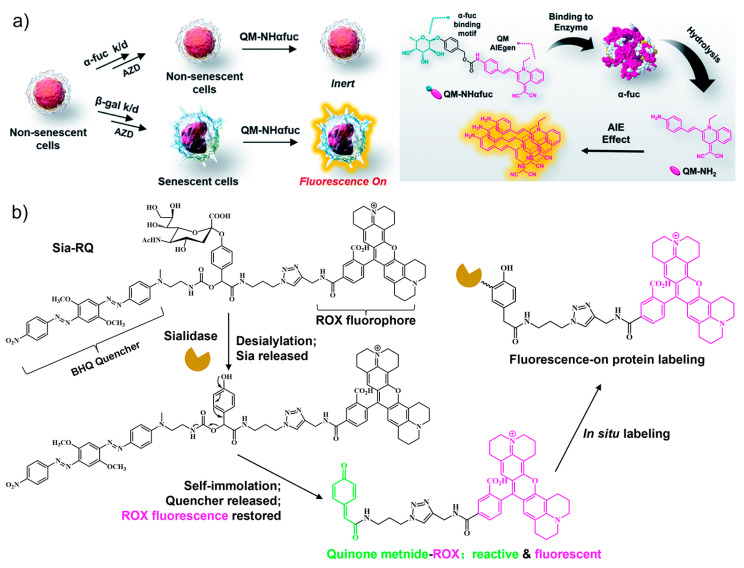
(**a**) The α-L-fucosidase (α-fuc)-activatable probe **QM-NHαfuc** was synthesized with an α-fucopyranoside group and an AIE fluorophore. The **QM-NHαfuc** was cleaved by α-fuc and able to identify senescent cells lacking β-Gal expression. Reprinted with permission from [65]. Copyright 2021 Royal Society of Chemistry; (**b**) an activity-based fluorescent sensor (Sia-RQ) synthesized from sialic acid as the targeting unit, a blackhole fluorescence quencher (BHQ), and a rhodamine-X (ROX) fluorophore. The fluorescence can be turned on by sialidase expressed in senescent cells. Reprinted with permission from [66]. Copyright 2018 Royal Society of Chemistry.

As a lysosomal acid hydrolase, α-fuc catalyzes the hydrolysis of *O*- and *S*-glycosyl compounds that contain a fucoside group, such as glycoproteins and glycolipids [64]. Based on the specific interaction between α-fuc and the substrate α-L-fucoside, targeted sensors were designed by using the fucoside as the recognition unit. For example, an α-fucopyranoside group was employed as the putative α-fuc recognition group and conjugated with an aggregation-induced emission (AIE) fluorophore quinoline–malononitrile (QM), affording an enzyme activatable probe **QM-NHαfuc** (Figure 3a) [65]. Owing to the high hydrophilicity of the fucopyranoside group, the sensor is non-emissive in its native state. In contrast, the cleavage by α-fuc generates the hydrophobic product which undergoes aggregation to emit bright fluorescence. The working principle was verified by in-solution experiments and cell imaging assays. Based on these, **QM-NHαfuc** was used to track cellular senescence induced by various stimuli including replication, ROS, ultraviolet A, and drugs. Notably, **QM-NHαfuc** was found to be able to identify senescent cells lacking β-Gal expression, providing a novel approach to facilitate the screening of potential senolytic drugs.

Lysosome-associated sialidase is another hydrolase that is significantly upregulated in senescent cells. By recognizing terminal sialic acid (Sia) residues, sialidase cleaves Sia from substrates such as glycoproteins and glycolipids [67,68]. Guided by this, Sia was used as the targeting unit for the design of an activity-based wash-free fluorescent sensor (Sia-RQ) [66]. Sia-RQ stays in a fluorescent-quenched status due to the presence of a blackhole fluorescence quencher (BHQ) paired with a rhodamine-X (ROX) fluorophore (Figure 3b). In palbociclib-induced senescent Huh-7 cells, Sia-RQ reacts with sialidase to release a reactive dye quinone intermediate, which further covalently labels sialidase to produce a strong fluorescence signal. The results from Sia-RQ manifest a significant upregulation of lysosomal sialidase activity during senescence and evidence the usefulness of sialidase as a novel biomarker for imaging senescence.

**Figure 4:**
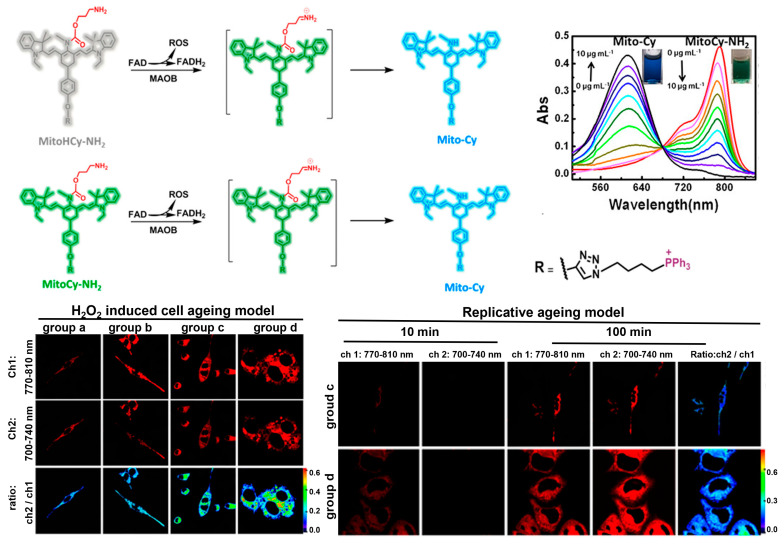
The fluorescence off–on sensor MitoCy-NH_2_ and ratiometric sensor MitoHCy-NH_2_, for synergistic imaging of MAO-B in senescent cells induced by H_2_O_2_ and replicative ageing. Reprinted with permission from [76]. Copyright 2018 American Chemical Society.

As the powerhouse of cells, mitochondria play central roles in thermogenesis and cellular metabolism where a diverse range of enzymatic reactions are involved [69,70,71]. Monoamine oxidases (MAOs) are mitochondrial enzymes that catalyze the oxidation of bioamines to the corresponding aldehydes, while producing ROS [72,73]. MAOs exist in two isomers, MAO-A and MAO-B, which have been revealed to be upregulated in many senescence-associated diseases, such as cardiovascular dysfunction, neurodegenerative diseases, and metabolic disorders [74,75]. Synthetic fluorescent probes that target MAOs can accurately assess the enzyme activity in biosystems, and thus enable sensitive detection and imaging analysis of senescent cells. Chen, Yu, and co-workers [76] designed two NIR fluorescent probes, MitoCy-NH_2_ and MitoHCy-NH_2_, for synergistic imaging of MAO-B in senescent cells and senescent mice (Figure 4). MitoHCy-NH_2_ is an off–on probe with fluorescence activatable by MAO-B, while MitoCy-NH_2_ exhibits ratiometric fluorescence, offering the opportunity to quantitatively access the activity of MAO-B in senescent cells. Directed by the cationic triphenylphosphine group, both probes are localized to mitochondria to target MAO-B in HepG2 cells. In H_2_O_2_-induced senescent HepG2 cells, MitoCy-NH_2_ can directly release fluorophores via a β-elimination reaction with MAO-B, leading to a ratiometric fluorescence response. In contrast, the activation of MitoHCy-NH_2_ requires the joint participation of MAO-B and ROS (O_2_^•−^) and synergistic response to MAO-B and ROS generated by the enzymatic reaction in the senescent HepG2 cells. Based on the critical role of MAO-B in neurodegenerative diseases, the probes were expected to be useful for the diagnosis of neurodegenerative diseases.

## 3. Targeted Sensing Senescence with Antibody-Functionalized and Antibody-Mimetic Nanoparticles

Antibody–antigen binding directed by a set of non-covalent interactions including electrostatic interactions, hydrogen bonds, van der Waals forces, and hydrophobic interactions, is one of the most important molecular recognition events in nature [77]. Owing to their high affinity and specificity, antibodies have been extensively employed as targeting moieties to guide the detection and regulation of various species ranging from metal ions to biomacromolecules and pathogens [78,79]. The modification of antibodies with fluorescent agents has been demonstrated as an important entity for imaging target molecules in biological samples, allowing the development of well-known assays such as immunofluorescence staining [80,81]. However, direct applications of antibodies for target tracking in living systems remain a challenging task, due to the immunogenicity, poor penetrability, and fragile nature of antibodies. Thanks to developments in molecular and material science, effective approaches have been established to enable the applications of antibodies in living cells and in vivo. In terms of fluorescence imaging of cellular senescence, antibodies were used in combination with nanoparticles. Since nanoparticles can facilely accumulate in lesion sites in the body and be internalized into cells [82], antibodies thus can be carried to exert molecular recognition effects to imaging senescent-related processes in living biosystems.

**Figure 5:**
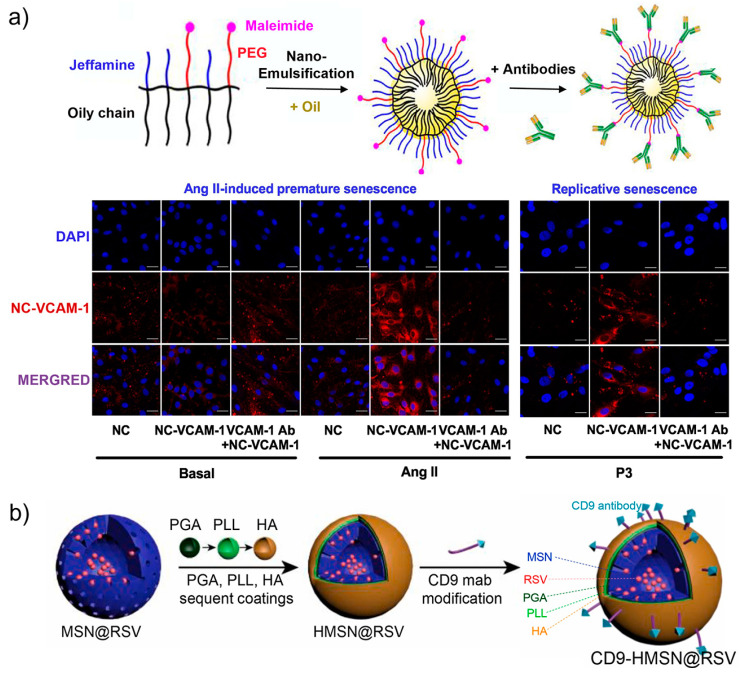
(**a**) Design and preparation of VCAM-1 antibody-decorated core-shell nanosensors (NC-VCAM-1) for imaging Ang II-induced and replicative senescent endothelial cells. Reprinted with permission from [84]. Copyright 2021 Elsevier; (**b**) Construction of antibody-decorated mesoporous silica nanoparticles with surface modification of CD9 antibody and capping with hyaluronic acid (HA), poly (L-lysine hydrochloride) (PLL), and methoxy-poly (ethylene glycol)-block-poly (L-glutamic acid sodium salt) (PGA) for targeting senescent foamy macrophages and senescent cells. Reprinted with permission from [85]. Copyright 2021 Elsevier.

As a hallmark of cardiovascular diseases, endothelial dysfunction is known to be accompanied with endothelial senescence [83]. Therefore, targeting and monitoring endothelial senescence has become crucial for the prevention of related diseases. Based on the increased expression of Vascular Cell Adhesion Molecule 1 (VCAM-1) on cell membranes, Schini-Kerth and co-workers constructed antibody-decorated core-shell nanosensors (NC-VCAM-1) to image senescent endothelial cells [84]. The lipid nanocarriers were prepared from an amphiphilic polymer and assembled via an ultrasonication method. Highly lipophilic dyes were loaded to the cores of the nanocarriers to provide fluorescent signal, while the hydrophilic surface was grafted with VCAM-1-targeting antibody via the maleimide groups. As shown in Figure 5a, the imaging performance of the nanocarriers were investigated with the Angiotensin II (Ang II)-induced senescent models. Compared with healthy endothelial cells, significant fluorescence was detected in premature senescent cells and replicative senescent cells as indicated by the antibody-functionalized nanosensors. Notably, the fluorescence signal was found to be dependent on the size of the nanoparticles and the loading of antibodies. The highest fluorescence signal was observed for nanocarriers below 100 nm in size for the formulation with an antibody concentration of 4.56 μg/mL. The VCAM-1-targeting fluorescent nanocarriers are promising for the early detection of senescent endothelial cells, and also may provide a means for preventing pro-senescent endothelial responses by localization of endothelial senescence.

CD9, a member of the tetraspanin family, has been generally known for its participation in numerous biological activities, such as cell migration, adhesion, and communications [86,87,88]. In recent years, CD9 was found to play a role in cellular senescence by the involvement in the phosphatidylinositide 3 kinase-AKT-mTOR-p53 signal pathway [89]. According to this, CD9 antibody-decorated mesoporous silica nanoparticles were fabricated for targeting senescent foamy macrophages and senescent endothelial cells (Figure 5b) [85]. In addition to antibody functionalization, the nanoparticles were coated with hyaluronic acid (HA), poly (L-lysine hydrochloride) (PLL), and methoxy-poly (ethylene glycol)-block-poly (L-glutamic acid sodium salt) (PGA). HA-capping was designed to introduce hyaluronidase-responsive release of payloads, while the PGA and PLL layers were used to enhance the stability, decrease the reticuloendothelial system uptake, inhibit plasma protein opsonization, and prolong the circulation period of the nanoparticles. Further loading of the silica nanoparticles with Cy 5.5 allows fluorescence tracking the targeting ability and distribution. In the senescent cell model stimulated with oxidized high-density lipoprotein, the antibody-modified nanoparticles were efficiently taken up by the cells. The CD9-targeting ability further enabled the in vivo delivery of the nanomaterials to the senescent atherosclerotic plaques, which is promising for developing an accurate theranostic approach for atherosclerosis.

**Figure 6:**
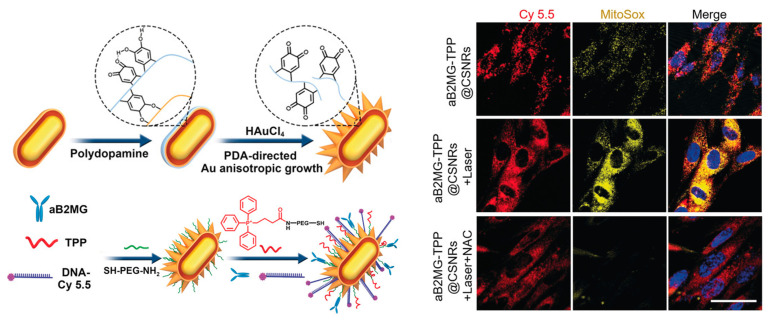
Design of the core–shell spiky nanorods (CSNRs) by the modification of gold nanorods with anti-beta 2 microglobulin (aB2MG) antibody, mitochondria-targeting TPP, and fluorescent DNA-Cy 5.5, and its application for imaging senescent cells. Reprinted with permission from [91]. Copyright 2020 Wiley.

Beta-2 microglobulin (B2M) has been revealed to be preferentially expressed in senescent cells and validated as a potential maker of senescence [90]. Based on this fact, antibodies against B2M were employed to functionalize nanomaterials for targeting B2M in cells and in situ tracking senescence. Kuang, Sun, and co-workers constructed a plasma core–shell spiky nanorods (CSNRs) whose surface was modified with anti-beta 2 microglobulin (aB2MG) antibody [91]. In addition, triphenylphosphonium (TPP) was also modified on the particle surface, aiming at further localizing to mitochondria. To verify the targeting ability of the nanomaterials, the as-prepared aB2MG-TPP@CSNRs were modified with Cy 5.5-tagged DNA to fluorescence track the distribution. As shown in Figure 6, the imaging results demonstrate the effective internalization and targeted accumulation of aB2MG-TPP@CSNRs in the mitochondria of senescent cells. Moreover, due to the plasmonic effect of the CSNRs, NIR-induced mitochondrial damage occurred, leading to senescent cell apoptosis. In vivo targeted and eliminating senescent cells were also achieved in a mouse model, manifesting the effectiveness of the multifunctional nanocomposite by using B2M as the target protein. Motivated by this, smart nanomaterials decorated with antibodies for targeting cellular senescence have continuously emerged [92,93]. In a research work from the same group, chiral Cu_x_Co_y_S nanoparticles were found to have different cell internalization abilities, with D-form showing much higher cellular uptake efficiency. Through modification of D-Cu_x_Co_y_S with B2M antibody, selective recognition of senescent cells was achieved in vitro and in vivo, which further facilitated targeted elimination of senescent cells under a magnetic field and NIR light.

Molecularly imprinted polymers (MIPs) prepared via the molecular imprinting technique are called artificial antibodies or synthetic receptors [94,95,96,97,98,99]. Compared with natural antibodies, MIPs show attractive merits including tailor-made versatility, easy-to-synthesize, reusability, and high stability even in harsh conditions [97]. To date, MIPs have been tailored toward various targets covering small organic molecules to proteins and cells, and contributed to the fields of separation, biosensing, bioimaging, and theranostics [98]. By using B2M as the template, MIPs have been fabricated for the recognition of this senescence marker protein with high affinity and selectivity [100,101]. The incorporation of fluorescence signal to MIPs can be achieved by compositing with fluorescent dyes or nanomaterials, offering the opportunities to image B2M and track senescence in biological systems.

One remaining challenge in MIPs is the preparation of effective imprinting sites for biomacromolecules such as proteins. Liu and co-workers reported a novel approach, named reverse microemulsion-confined epitope-oriented surface imprinting and cladding (ROSIC), for engineering antibody-mimetic nanoparticles with tunable monodispersed size and specific targeting capability toward B2M protein [102]. The C-terminal epitope of B2M was used as the template instead of the whole protein for the preparation MIPs. By using the ROSIC approach, the B2M C-terminal epitope-imprinted MIPs obtained a high affinity toward B2M with a *K_d_* value of 1.63 ± 0.34 × 10^–8^ M, which is close to the binding ability of monoclonal antibody. Further, controllable construction of such MIP shells on various functional nanoparticles was achieved. By using fluorescent quantum dots (QDs) as the core, the QD@cMIPs were applied for targeted imaging of proteins in living cells and in vivo. Considering the presence of glycolation sites in the C-terminal of B2M, the same group developed a new method termed boronate affinity-anchored epitope-oriented surface imprinting and cladding [103]. Taking advantage of the well-characterized affinity binding between boronic acids and cis-diol moieties, the as-prepared MIPs displayed enhanced affinity to target with a *K_d_* value of 1.12 × 10^−9^ M. By doping the MIP nanoparticles with fluorescein isothiocyanate, specific recognition, and track of target B2M, cellular senescence can be expected.

MIP particles from batch preparation are usually highly heterogenous in binding ability. In order to select MIPs with optimal targeting performance, a solid-phase approach was proposed, in which the template molecules are immobilized on a solid support during the polymerization [104]. Meanwhile, the template-immobilized support also acts as an affinity medium for the affinity screening of MIPs. By using this approach, Macip, Canfarotta, and co-workers synthesized and identified nanoMIPs targeting against the senescence membrane marker B2M [105]. A peptide fragment (amino acids 101–115) of B2M was chosen as the template for the preparation and screening of nanoMIPs. The optimized nanoMIPs were tagged with fluorescein to enable fluorescence imaging of B2M. NanoMIPs preferentially aggregated in senescent cells and specifically bound to the membrane on the tetracycline-induced senescent bladder cancer cells. For in vivo studies, the nanoMIPs were labeled with a DyLight 800 NHS ester and injected intravenously into mice. Strong fluorescence was imaged in 11-month-old mice, while a negligible signal was detected in the young mice after the same treatment, demonstrating the effectiveness of the B2M-targeting nanoMIPs for recognizing and tracking senescent cells.

## 4. Peptide-Guided Fluorescence Imaging of Cellular Senescence

Peptides, built from amino acids, participate in various cellular processes and are some of the most important biomolecules in living systems [106]. As a small version of proteins, peptides show unique features of flexible conformation, high stability, and adjustable affinity [107,108,109,110]. Facilitated by the development of solid-phase synthesis strategy, chemical synthesis of peptides of desirable sequences can be easily achieved [111,112]. Through rational designing and screening, artificial peptides can be tailored towards proteins of interest [113,114,115]. Both naturally occurring peptides and synthetic peptides have been employed as targeting units for the construction of fluorescent sensors, which have shown outstanding performance in targeted investigation of biological events with high affinity and high selectivity [116,117]. In recent years, peptides were also employed as affinity ligands for the development of senescence-targeting sensors, which contributed to the understanding of the complicated processes of this biological phenomenon.

**Figure 7:**
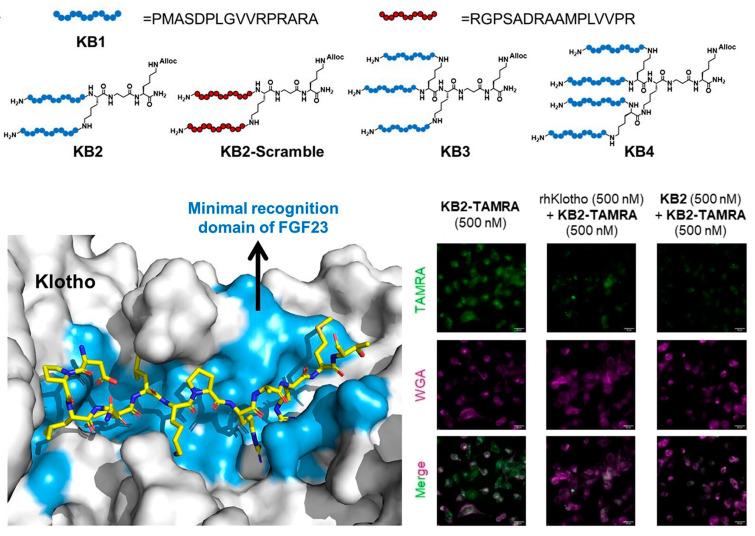
Branched multimeric peptide-based sensors with enhanced affinity to α-Klotho for imaging detection of senescent cells. Reprinted with permission from [120]. Copyright 2023 Wiley.

Protein–protein interactions (PPIs) control a wide range of biological activities, including signal transduction, cell proliferation, differentiation, and senescence, and have emerged as new potential targets for the diagnosis and therapy of diseases [118]. However, targeting and modulating PPIs are generally difficult, and as a result, PPIs were considered as “undruggable” [119]. Capable of mimicking the binding interfaces in protein, peptides have become attractive for targeting PPIs showing great potential as molecular tools for investigating and interfering protein functions. Recently, peptide-based agents were developed by Pentelute, López, and co-workers toward the binding interfaces between an aging-related protein α-Klotho and fibroblast growth factor 23 (FGF23) [120]. A peptide (**KB1**) mimicking the C-terminal of FGF23 was selected as the starting agent for the design of branched affinity ligands. Compared with the parent monomer **KB1**, the obtained dimeric, trimeric, and tetrametic peptides show 27.7-, 65.7-, and 26.1-fold improvements in affinity toward α-Klotho, respectively. Due to the high affinity and selectivity, the dimeric peptide **KB2** was tagged with fluorophore. As shown in Figure 7, the peptide sensor **KB2-TAMRA** successfully enters cells and labels target protein α-Klotho in living cells with bright fluorescence, showing the promises of the peptide-based sensors for imaging and detecting aging-related biological events in physiological settings.

**Figure 8:**
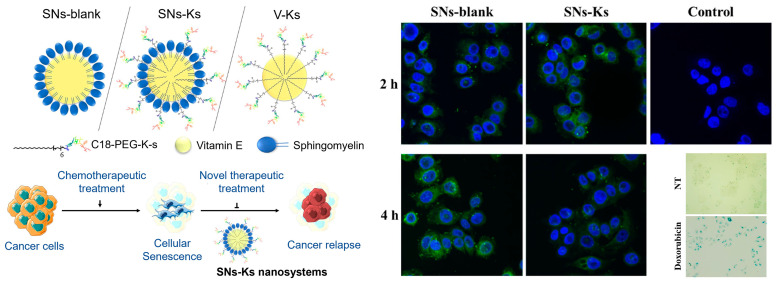
CD47-targeting peptide was integrated on the surface of the nanoemulsions composed of sphingomyelin for the imaging of senescent cells. Reprinted with permission from [121]. Copyright 2022 Elsevier.

Despite of the diverse advantages of peptide-based targeting strategies, in vivo applications of peptides still face challenges including limited half-life, low bioavailability, and fast renal clearance [108,114]. To address these, nanomaterial-based delivery systems have been introduced. Aiming at the delivery of peptides for targeting cellular-senescence, biodegradable and biocompatible nanoemulsions composed of sphingomyelin were employed as the carrier [121]. A peptide specifically recognizing CD47 overexpressed on the surface of senescent cells was derived with a polyethylene glycol-modified stearic acid carbon chain (C18), so as to enable the integration and display of the peptide on the surface of the nanoparticles. The resultant nanosystem (SNs-Ks), with an average size of ~100 nm, exhibited stability in bio-relevant media. As shown in Figure 8, compared with free peptide, SNs-Ks was efficiently internalized into MCF-7 cells with improved hemocompatibility. By taking advantage of these features, SNs-Ks could effectively target adriamycin-induced senescent breast cancer cells with the guidance from peptide-CD47 recognition.

In the past decade, an increasing number of proteins have been identified to play key roles in cellular senescence, which is followed by the development of peptide ligands with high affinity and selectivity to them. Apart from protein targets, other senescence-related biomolecules such as lipids were also identified [122,123] and employed as molecular targets for recognition by affinity peptides. For example, cardiolipin, an important component of the inner mitochondrial membrane, can restore mitochondrial bioenergy, remodel mitochondrial cristae structure, and restore organ function during senescence [124]. By using cardiolipin as the target, a class of cell-penetrating aromatic cationic tetrapeptides were discovered [125]. Directed by the specific binding toward cardiolipin in the mitochondrial membrane, the peptides exhibit functionalities for reducing ROS, remodeling mitochondrial cristae structure, and repairing cellular structure during senescence. By using these peptides as the targeting units, fluorescent sensors for imaging and tracking mitochondria in senescent cells can also be expected.

## 5. Dual-Targeting Fluorescent Sensors for Precisely Tracking Cellular Senescence

Cellular senescence is characterized by high heterogenicity and the expression of complex phenotypes [2,7]. Many cellular and molecular signatures have been exploited as senescent markers, however, none of them are exclusively expressed in senescent cells [4]. In view of this, the combination usage of biomarkers emerges as an effective way to achieve specific and accurate recognition of senescent cells. Thus, rational design and synthesis of the sensor become crucial to acquire simultaneous responses to multiple biomarkers. In recent years, fluorescent sensors with dual-targeting ability have been reported, which show improved selectivity for targeted investigation of senescent cells.

**Figure 9:**
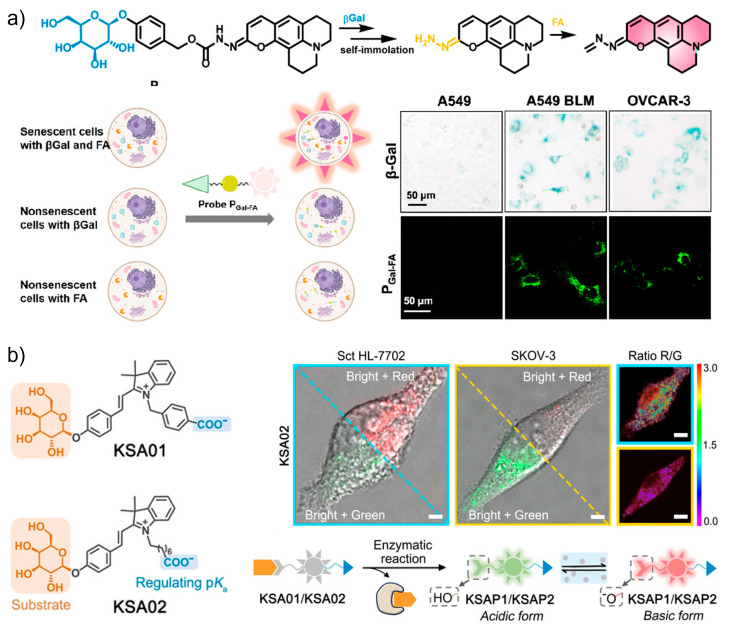
(**a**) The tandemly activated fluorescence probe (**P_Gal-FA_**) with dual-targeting ability toward β-Gal and formaldehyde (FA) for the highly selective discrimination of senescent cells from β-Gal-positive non-senescent cells. Reprinted with permission from [126]. Copyright 2022 American Chemical Society; (**b**) two-dimensional design of pH-sensitive and SA-β-Gal-targeting fluorescent probes (**KSA01** and **KSA02**) for the precise tracking of senescence with ratiometric fluorescence. Reprinted with permission from [23]. Copyright 2021 Wiley.

Although β-Gal has been extensively employed as a marker for senescence detection, this enzyme is also expressed in cancer cells and maturing tissue macrophages. To discriminate senescent cells from β-Gal-positive non-senescent cells, Li, Zhang, and co-workers designed a tandemly activated fluorescence probe (**P_Gal-FA_**) that showed dual responses to β-Gal and formaldehyde (FA) as markers [126]. The reason for choosing FA as the second marker is based on the fact that this oxidative stress-related metabolite contributes to age-related pathologies, and is a marker independent of the β-Gal signaling. To provide FA responsiveness, a hydrazonate group was modified to the coumarin core. The tandem responsiveness was achieved by the hydrolytic reaction with β-Gal to release the self-immolative linker *p*-hydroxybenzyl alcohol which undergoes spontaneous elimination reaction affording a free hydrazonate group to nucleophilically react with FA. By using this pathway, the fluorescence signal can only be triggered by the co-existence of β-Gal and FA. As shown in Figure 9a, during the application of **P_Gal-FA_**, simultaneous detection of the biomarker combination in the same live cell was realized. Compared with traditional single-biomarker-based probes, improved selectivity toward senescent cells can be obtained by the combination usage of two biomarkers. The reliability of the double-check strategy was further demonstrated by the fluorescence imaging of senescent cells in bleomycin-induced pulmonary fibrosis tissues.

The discrimination of SA-β-Gal from other endogenous β-Gal is an effective way to ensure the accuracy of senescence targeting, but remains challenging. As a lysosomal enzyme, the activity of SA-β-Gal is pH-dependent, meanwhile the pH in lysosomes is known to increase as senescence progresses. With this guidance, a two-dimensional design was innovated to construct fluorescent probes (**KSA01** and **KSA02**) for the precise tracking of senescence (Figure 9b) [23]. **KSA01** and **KSA02** were, respectively, developed from two merocyanine-based fluorescent dyes **KSAP1** (*pK_a_* 5.8) and **KSAP2** (*pK_a_* 6.2) with ratiometric responses to pH. The attachment of the recognition unit β-D-galactosyl through the glycosidic bond affords the final non-fluorescent two-dimensional probes **KSA01** and **KSA02**. The two-step sensing mechanism includes the binding and cleavage reaction with β-Gal, which releases the pH-sensitive fluorophores **KSAP1** and **KSAP2**. Then, the two dyes respond to lysosomal pH with green fluorescence under an acidic environment and red fluorescence under more basic conditions. As indicated by the probe, senescent cells emitted bright red fluorescence (λ = 562 nm), whereas non-senescent ovarian cancer cells showed green fluorescence (λ = 534 nm) after the same treatment. Their results manifest the specificity of the probe and effectiveness of the two-dimensional strategy for sensitively differentiating senescent cells.

**Figure 10:**
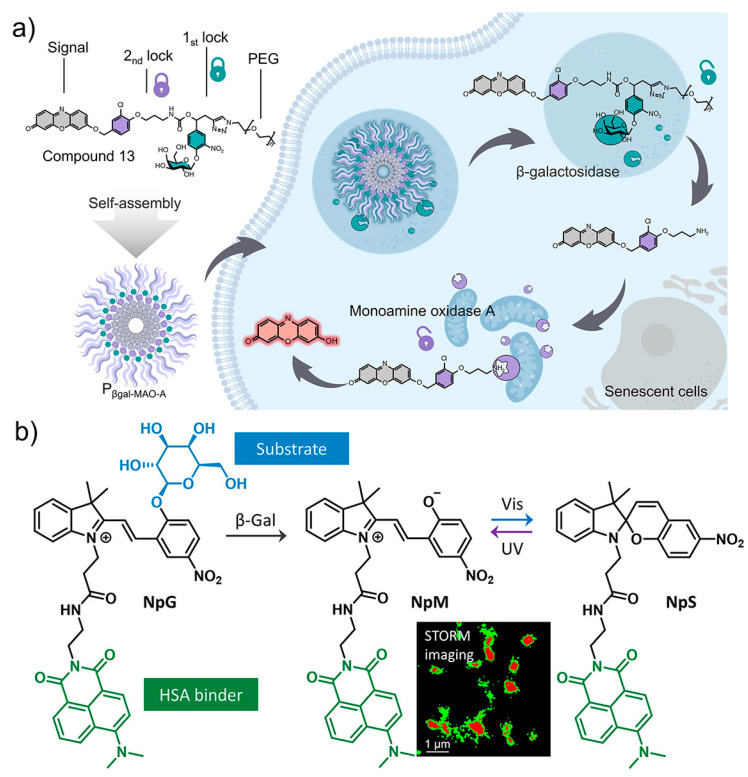
(**a**) The design and fluorescence sensing mechanism of **P_βgal-MAO-A_** with dual-targeting ability to SA-β-Gal and MAO-A, producing double-locked fluorescence for specific imaging of senescent cells. Reprinted with permission from [127]. Copyright 2023 American Chemical Society; (**b**) The photochromic fluorescent probe (**NpG**) with affinity binding to β-Gal and human serum albumin, affording switchable fluorescence for the super-resolution imaging of cellular senescence. Reprinted with permission from [128]. Copyright 2020 American Chemical Society.

As discussed above, after the identification of SA-β-Gal as the first molecular target for senescence, a growing number of enzymes and proteins were proposed as potential markers. The combination of SA-β-Gal with other protein-based makers gave rise to the novel dual-targeting fluorescence sensors for precise imaging [127]. By using SA-β-Gal and MAO-A as the two targets, Liu and co-workers designed and synthesized a two-parameter recognition fluorescent probe **P_βgal-MAO-A_** (Figure 10a). The molecular structure of **P_βgal-MAO-A_** consists of a galactose unit, a signal unit resorufin, and the MAO-A-recognition moiety. The fluorescence of the probe is double locked by the β-Gal-responsive and MAO-A-responsive units. Therefore, only senescent cells co-expressing the two enzymes can be lit up with bright fluorescence, providing an accurate visualization method for tracking cellular senescence in biological systems.

Imaging enzymatic activity with spatial/temporal resolution is highly desirable for investigating their biological function with high precision, where fluorescent probes with switchable signals are usually required. By using β-Gal and human serum albumin (HSA) as the target, a photochromic fluorescent probe (**NpG**) was designed for super-resolution imaging of β-Gal during senescence (Figure 10b) [128]. Driven by the binding with HSA, a probe/protein hybrid **NpG@HSA** is formed with visualizable spiropyran fluorescence. Efficient cellular uptake was determined for **NpG@HSA**, which is favorable for imaging β-Gal activity inside cells. In the presence of β-Gal, the galactose-caged fluorescent at 620 nm was released due to the cleavage of the galactose unit and the release of **NpM@HSA**. The photoisomerization of merocyanine unit in **NpM@HSA** can produce ON/OFF photoblinking which can be used for stochastic optical reconstruction microscopy imaging. Visualization of changes of β-Gal activity and its subcellular distribution was realized with nanoscale precision, providing more detailed information on lysosomal β-Gal in senescent cells.

**Table 1:** Summary and compare of molecularly targeted fluorescence sensors for senescent imaging.

Types of Markers	Recognition Elements	Probes	Performance Constants	Fluorescent Reporters	Detection Approach and Signal	Imaging Applications	Refs
β-Gal and pH	β-D-galactose	**KSA01** **KSA02**	LOD = 8.3 × 10^−3^ U mL^−1^ LOD = 6.8 × 10^−3^ U mL^−1^	Merocyanine	Ratiometric Turn-on	Cells Tissues	[23]
β-Gal	Acetylated galactose	**AcGQCy7**		Quinone-cyanine-7	Turn-on	Cells	[30]
	β-D-galactopyranoside	**βGal-1**	LOD = 4.62 ± 0.46 × 10^−5^ U mL^−1^	6-amino-styryl-benzothiazole	Turn-on	Cells	[33]
	β-D-galactose	TC-gal	LOD = 8.4 × 10^−5^ U mL^−1^ *K_m_* = 18.4 μM	Tetraphenylethylene and coumarin	Turn-on	Cells	[34]
	β-D-galactose	**TPh-PyBz-β-gal**	LOD = 0.22 U mL^−1^	Tetraphenylethylene	Turn-on	Cells	[35]
	Acetylated galactose	**AHGa**		Naphthalimide	Turn-on Two-Photon	Cells Tumors (in vivo)	[38]
	β-D-galactopyranoside	**MB-** ***β*** **gal**	LOD = 0.156 mU mL^−1^	Methylene blue	Turn-on NIR	Cells	[44]
	Acetylated galactose	**NBGal**	LOD = 2.33 U mL^−1^	Nile blue	Turn-on NIR	Cells Tumors (in vivo) Tissues	[45]
	β-D-galactopyranoside	DSL-Gal	*K_m_* = 78.75 μM *V_max_* = 4.17 μM s^−1^ LOD = 0.036 U mL^−1^	Dual-state luminophores	Turn-on NIR	Cells Tissues Tumors (in vivo)	[47]
	β-D-galactose	**Gal-HCy-Biotin**	*K_m_* = 4.60 μM^–1^ s^–1^ LOD = 3.7 × 10^−3^ U mL^−1^	Hemicyanine dye	Turn-on NIR	Cells Tumors (in vivo)	[48]
	β-D-galactose	**BOD-L-** ***β*** **Gal**	*K_m_* = 34.6 μM *V_m_* = 13.52 μM s^−1^ LOD = 0.014 U mL^−1^	Boron dipyrromethene	Ratiometric Turn-on NIR	Cells Tissues Senescent mice (in vivo)	[49]
	β-D-galactose	**KSL0608-Se**	LOD = 8.96 × 10^−2^ U mL^−1^	Dicyanomethylene-4H-pyran	Turn-on NIR	Cells Senescent mice (in vivo)	[50]
α-L-fucosidase	α-fucopyranoside	**QM-NH** **α** **fuc**	LOD = 1.0 × 10^−2^ U mL^−1^	Quinoline-malononitrile	Turn-on	Cells Tumors (in vivo)	[65]
sialidase	Sialic acid	Sia-RQ		Rhodamine-X	Turn-on	Cells	[66]
MAO-B	Propanamide	MitoCy-NH_2_ MitoHCy-NH_2_	*K_m_* = 10.13 ± 0.28 μM *V_max_* = 3.55 nmol mg^–1^ min^–1^	Heptamethine cyanine	Turn-on Ratiometric NIR	Cells Brains (in vivo)	[76]
VCAM-1	Antibody	NC-VCAM-1 (nanosensors)		NR668 Cy 5.5-TPB		Cells	[84]
CD9	Antibody	CD9-HMSN@RSV		Cy 5.5		Cells Tissues	[85]
B2MG	Antibody	aB2MG-TPP@CSNRs		Cy 5.5		Cells Senescent mice (in vivo)	[91]
B2MG	Antibody	D-Cu_x_Co_y_S (NPs)		Penicillamine		Cells	[92]
B2MG	Antibody	UAuTe(NPs)		Cy 5.5		Cells Tissues Senescent mice (in vivo)	[93]
B2MG	Antibody mimetic MIP	QD@cMIPs	*K_d_* = 1.63 ± 0.34 × 10^–8^ M	FITC		Cells Tumor (in vivo)	[102]
B2M	Antibody mimetic MIP	B2M nanoMIPs		DyLight 800 NHS ester, Alexa Fluor 647		Cells Senescent mice (in vivo)	[105]
α-Klotho	Peptide (**KB2**)	**KB2-TAMRA**	*K_d_* = 90 ± 31 nM	TAMRA	Turn-on	Cells	[120]
CD47	Peptide (4N1Ks)	SNs-Ks(NPs)		TopFluor^®^-SM	Turn-on	Cells	[121]
β-Gal and FA	β-D-galactose and hydrazonic acid	**P_Gal-FA_**		Coumarin-hydrazonate	Turn-on	Cells Senescent mice (in vivo)	[126]
β-Gal and MAO-A	β-D-galactose and propylamine replaces m-chlorophenol	**P** **_β_** **_gal-MAO-A_**		Compound 6	Turn-on	Cells	[127]
β-Gal and HSA	β-D-galactose and merocyanine	**NpG**	*K_m_* = 4.34 μM *V_max_* = 23.37 nM s^−1^ LOD = 6.1 × 10^–4^ U mL^−1^ *K_d_* = 27.34 ± 1.93 μM(HSA)	Merocyanine	Turn-on	Cells	[128]

## 6. Summary and Perspectives

Cellular senescence, a permanent state of cell cycle arrest, is a main feature of aging, and is also closely related with the origin and progress of a range of diseases. In the past decades, senescence has gained remarkable attention as a promising target for the diagnosis and treatment of various age-related diseases, where the identification, monitoring, and chemical regulation of senescent cells has become fundamental and crucial. Tremendous research efforts have been made toward the development of chemical tools for the investigation and modulation of cellular senescence at the molecular level. Motivated by the rapid development of the field, we herein reviewed the recent progress in targeted fluorescence sensors for tracking and detecting cellular senescence. Particular attention was paid to the targeting groups that specifically recognize senescence-related biomarkers and their cooperative action with fluorescent units during sensing. A majority of currently available sensors are designed toward SA-β-Gal, since this enzyme is the first marker used for the identification of senescent cells. As more secreted and/or intracellular proteins were proposed as potential biomarkers, various targeting ligands with high affinity and specificity were designed, including carbohydrates, antibodies, and peptides. Apart from molecular recognition, these targeting elements are also used as responsive modules to control the fluorescent signals, which is realized by the conjugation with fluorophores via cleavable or switchable bonds. Triggered by senescence-related enzymes and proteins, the rationally designed sensors display off–on or ratiometric fluorescence, enabling highly selective imaging analysis of marker molecules in senescent cells. The integration of affinity ligands with NIR fluorophores or nanoparticles, in situ localization, and real-time monitoring of senescence under different stimuli such as DNA damage, oxidative stress, and chemotherapy have been achieved in living cells and in vivo. By using these fluorescent sensors, important information on dynamic changes of molecular markers during senescence has been obtained, which can be helpful for in-depth understanding of this biological process.

Although great advances have been achieved for the molecularly targeted sensors, there are still challenges that remain to be addressed. As the phenotype associated with cellular senescence is highly variable, heterogeneous, and plastic, currently used markers are not exclusive to senescence, which brings difficulties to identifying senescent cells accurately. Novel and more specific biomarkers of senescence need to be discovered and identified through advanced proteomics and other techniques, but it is not always easy. To tackle this, the combination of two markers has been proposed instead of the usage of a single hallmark, which can reinforce the targeting accuracy. Further combinations of multiple biomarkers for the design of fluorescent sensors can be expected. The recognition and analysis of different targets can not only significantly improve the specificity of imaging, but also provide useful information to study the heterogenicity of senescence. On the other side, targeting multiple markers by one sensor is challenging, both in molecular design and chemical synthesis. In addition to the heterogeneity of markers, senescent cells also exhibit different phenotypes as the senescence process progresses. Particularly, in order to detect the markers in the early senescence stage at a lower concentration level, higher requirements are put forward for the detection limit and sensitivity of fluorescence sensors. This requires not only the development of targeted units with higher affinity and specificity, but also novel fluorescent molecules with high quantum yields, large Stokes shifts, and high photostability. In the late stage of senescence, accumulation and persistence of senescent cells can induce a chronic low-grade inflammatory state which is usually associated with aging and chronic diseases. Therefore, multifunctional sensors integrating molecular recognition, fluorescent detection and regulation, and removal of cellular senescence are also attractive. Such theranostic sensors would allow real-time monitoring of the changes of markers while exerting therapeutic effects, providing immediate feedback of the treatment. Moreover, to meet the demand for in vivo imaging and long-term tracking, fluorescent sensors with high penetrability, stability, biosafety, and unique optical properties are strongly demanded. Although there have been NIR sensors, sensors operated in the NIR-II window have rarely been reported yet. In addition, not only limited to fluorescence imaging, but also new imaging methods are being developed and combined, such as photoacoustic imaging, magnetic resonance imaging, as well as multimodal imaging strategies, which show higher selectivity, sensitivity, and spatiotemporal resolution for visualization and tracking of senescence-related biological processes in vivo and in vitro. Senescence-derived exosomes are nano-sized extracellular vesicles that play important roles in biological processes. Senescent cells typically secrete more functional exosomes than proliferating cells and interact with other senescent and non-senescent cells. The detection and imaging of senescence-derived exosomes will provide a deep understanding of intercellular transport and communication in senescence, which is more difficult and challenging than that of senescent cells. This is not only due to the nanoscale size of these vesicles, but also because the senescence-derived exosomes are shielded by the vast number of normal exosomes. Different from the conventional exosomal markers that have been widely used, there is still a lack of clear markers for senescence-derived exosomes. Therefore, the screening of its markers and flexible design of fluorescence sensors are expected to achieve precise tracking and real-time visualization of senescence-derived exosomes. In addition to exosomes, senescent cells also secrete a plethora of factors, including growth modulators, pro-inflammatory cytokines, and chemokines. Detection of these senescence-related secretions using advanced fluorescence sensors will also help define senescence-based molecular signatures. Based on the available knowledge on the molecular design and sensing mechanism, engineering next-generation fluorescent sensors with unique and profound performance can be expected, which will greatly benefit the insights into the senescence-related life processes.
